# Recurrent Left-Sided Pleural Effusions in a Patient With Chronic Lymphedema

**DOI:** 10.7759/cureus.33167

**Published:** 2022-12-31

**Authors:** Kristin N Slater, Sohaib Ahmed, Uzoamaka Obodo, Sam Son, Fagunkumar Modi

**Affiliations:** 1 Internal Medicine, Lincoln Memorial University DeBusk College of Osteopathic Medicine, Harrogate, USA; 2 Internal Medicine, Nova Southeastern University Dr. Kiran C. Patel College of Osteopathic Medicine, Tampa, USA; 3 Internal Medicine, HCA Florida Citrus Hospital, Inverness, USA; 4 Pulmonology and Critical Care, HCA Florida Citrus Hospital, Inverness, USA

**Keywords:** hereditary lymphedema type 1, hereditary lymphedema, primary lymphedema, lymphedema, chronic lymphedema, milroy’s disease

## Abstract

Chronic lymphedema can lead to several long-term complications. The causes of lymphedema can be primary, due to a genetic source, or secondary to procedures, trauma, or other conditions. Primary hereditary lymphedema, as in the case of Milroy’s disease, is rare. Because of the condition's rarity, case reports mostly involve presentations to monitor for. Here we document a case of Milroy’s disease in a 70-year-old woman with recurrent left lung effusions.

## Introduction

Milroy’s disease is a rare genetic condition with a worldwide incidence of about 1/6000. It has an autosomal dominant inheritance [1-3] with a higher incidence in females [2]. It is a primary, hereditary source of lymphedema, largely caused by genetic mutations in FLT4/VEGFR3, which are involved in lymphangiogenesis [1-3]. FLT4/VEGFR3 abnormalities cause lymphatic aplasia or lymphatic collector dysfunction, thereby slowing the flow of lymph through defective nodes [1-2]. Symptoms of Milroy’s disease occur early, with some cases reporting early bilateral dorsal edema symptoms at birth [3]. Milroy’s disease often presents with bilateral swelling in differing distributions, but commonly affects the lower limbs and can affect all extremities [1-3]. Some complications of chronic lymphedema include infections/cellulitis [4], pleural effusions, septic arthritis, and intestinal lymphangiectasia [3]. Treatments are limited and often involve conservative measures such as compression to improve swelling and tissue oxygenation [5]. Normally, management is symptomatic, treating complications as and when they arise.

## Case presentation

A 70-year-old female presented to the clinic for the management of pleural effusions and chronic lymphedema. Her past medical history was remarkable for hyperlipidemia, heart failure, and Milroy's disease (hereditary lymphedema). She managed her conditions with azelastine as needed, triamterene nightly, torsemide twice weekly, furosemide as needed, sacubitril-valsartan twice daily, meloxicam three times daily, and loratadine daily. Her family history was significant for chronic lymphedema throughout her paternal side of the family including her father, aunt, uncle, and cousins. At the age of 15 years, she had been examined for increased leg swelling during her menstrual cycle. By the age of 18 years, she had been started on a thiazide to manage generalized edema. She had begun to use compression stockings and garments to alleviate some of the lymphedema symptoms and implemented dietary changes, limiting processed foods, and salts. In her 40s and 50s, she had begun to develop a series of hand infections secondary to lymphedema of the upper extremities, which had been managed with antibiotics. She developed heart failure four years ago. At that time, she had begun experiencing left-sided pleural effusions. The fluid buildup caused significant pressure, making her unable to walk two blocks without shortness of breath. This classified her functional status as New York Heart Association (NYHA) stage 3C. 

The patient presented to our office with a chief complaint of significant dyspnea and orthopnea requiring her to position herself upright during sleep to facilitate non-labored breathing. Her physical examination showed decreased breath sounds and dullness to percussion over the left lung. She had experienced multiple, recurrent pleural effusions on her left side. She reported having a total of eight thoracenteses to treat large recurrent left-sided pleural effusions over the last four years. The most recent thoracenteses of her left lung had been in July and October, yielding a pleural fluid output of 1750 mL and 1500 mL, respectively. Analysis showed no malignancy. Her presentation was one of chronic lymphedema and recurrent pleural effusions secondary to Milroy’s disease. As a genetic condition, Milroy’s disease does not have definitive treatment at this time. The management mostly involves treating symptoms as and when they arise. In our case, therapeutic thoracenteses were used to treat our patient’s recurrent pleural effusions.

## Discussion

Milroy’s disease (hereditary lymphedema, type 1) is a rare inherited condition resulting in impaired lymphatic drainage due to an FLT4/VEGFR3 gene mutation [1-3]. The disease presents most commonly with bilateral lymphedema of the lower legs but can affect all extremities [1-3]. Complications can arise, including pleural effusions, which in our patient’s case were large, recurrent, and left-sided. Treatments largely consist of symptom management and conservative measures such as compression [5]. Newer surgical techniques such as vascularized lymph node transfer (VLNT) with therapeutic lipectomy have been explored in moderate cases to improve lymphatic drainage [6]. But these reports record only small numbers of patients, likely due to the rarity of the condition. Our case documents a presentation of Milroy’s disease involving chronic lymphedema of all extremities with recurrent left-sided pleural effusions and a strong family history of primary chronic lymphedema. Our patient's early experiences also highlighted increased bilateral lower limb lymphedema during menstruation and recurrent hand infections secondary to her lymphedematous state. Infections and cellulitis are reported in the literature both in primary and secondary lymphedema [4]. To the best of our knowledge, increased bilateral lymphedema of the lower extremities during menstruation has not yet been reported. 

Our patient conveyed feelings of frustration over dismissal by doctors who did not fully understand her condition. Barriers to understanding Milroy’s disease are multifactorial. The rarity and lack of public knowledge and education regarding the condition partly explain the issue. If the condition does not fit the classic symptom profile comprising limited presentations that are taught in medical training (often shown as extreme presentations), it may not be considered a diagnostic possibility. Subsequently, the patient can be left to manage their own care and advocate for their own health in rare chronic conditions, such as Milroy’s disease. It was proposed that chronic lymphedema in all forms encounter medical barriers to care because of the ambiguity of the diagnostic criteria, the lack of cultural, social, economic, and scientific capital as well as a lack of media representations [7]. This complex combination of factors is linked to sociological interplay [7]. Unclear parameters and lack of clear objectives in treating lymphedema combined with limited known effective treatments also play a role [7]. Additionally, our patient reported experiencing dismissive attitudes from physicians due to her gender. Gender bias is known to exist in medicine, which can affect patient outcomes [8-9]. It is our sincere belief that this case report will contribute to the medical literature and literacy surrounding Milroy’s disease with the goal of optimizing treatments and patient care by promoting physician understanding of Milroy’s disease and its various presentations.

## Conclusions

Milroy’s disease is a rare condition with several presentations and complications. In a patient with recurrent large pleural effusions, chronic lymphedema, and a family history of chronic lymphedema, Milroy’s disease should be considered. This case documents an example of recurrent left-sided pleural effusions as a presentation of Milroy’s disease.
